# Supplementary material for: The role of genetic diversity in the evolution and maintenance of environmentally-cued, male alternative reproductive tactics
Source: BMC Evol Biol. 2019 Feb 18;19:58. doi: 10.1186/s12862-019-1385-4 (PMC6379956; doi:10.1186/s12862-019-1385-4)
Supplement: Supplementary file 1 — Table S2.1. – S2.5. Exemplar (1 from 5) of subsampled analyses performed from 30 randomly sampled individuals per subpopulation (female, fighter, scrambler) between rich and poor environments. Table S2.1. nSSR information on each locus. Table S2.2. Hardy-Weinberg statistics across environments and subpopulations. Table S2.3. Pairwise FST values for population differentiation. Table S2.4. Summary of hierarchical AMOVA. Table S2.5. Genetic differentiation between strategies (fighters and scramblers) compared to total population within that environment (rich and poor). (DOCX 32 kb) [file 12862_2019_1385_MOESM1_ESM.docx]

Additional File 1

Exemplar (from 5) of subsampled analyses performed from 30 randomly sampled individuals per subpopulation (female, fighter, scrambler) between rich and poor environments.

Table S2.1 nSSR information on each locus. Names, type of repetitive motif, size range of alleles (bp), primer sequences (forward - F, reverse - R), number of alleles (n_A_), annealing temperature (T_a_), primer concentration used in PCR amplification (Pc), and observed (H_O_) and expected (H_S_) heterozygosities, with corresponding p-values.

| **Locus** | **Nucleotide repeat** | **Size (bp)** | **Primer sequence** | **n_A_** | **T_a_ (°C)** | **Pc (μM)** | **H_O_** | **H_S_** | **p-value** |
| --- | --- | --- | --- | --- | --- | --- | --- | --- | --- |
| *Rrms18* | CATT | 130 – 143 | F: GCTTTCATTGTTGTACACCTC  R: ACAAACAGCAATGAGGTACAG | 4 | 53 | 3 | 0.197 | 0.490 | <0.001 |
| *Rrms34* | TGAA | 106 – 136 | F: AATAATGTTTCGCACTGAGAG  R: CAAGGTAGACCGTTACAGTGA | 10 | 53 | 15 | 0.713 | 0.759 | 0.064 |
| *Rrms40* | CACT | 85 – 118 | F: GTAATGGCCATGTCACTAGC  R: TTTGAGACTCGAAAGAAACAG | 9 | 53 | 10 | 0.255 | 0.550 | <0.001 |
| *Rrms44* | GAGT | 91 – 98 | F: CTATGTTGAAAAGGCATCAAT  R: GCAAAGTGTTGTTCACTCAAT | 3 | 51 | 15 | 0.391 | 0.357 | 0.088 |
| *Rrms72* | CATT | 128 – 142 | F: GAAATGTCAAAGACGAAAGTG  R: TTGAAGTGCGAAATTAGTCAT | 8 | 51 | 15 | 0.671 | 0.716 | 0.123 |
| *Rrms91* | GAGT | 84 – 92 | F: CTATGTTGAAAAGGCATCAAT  R: GCAAAGTGTTGTTCACTCAAT | 4 | 51 | 5 | 0.360 | 0.482 | <0.001 |

**Table S2.2.** Hardy-Weinberg statistics across environments and subpopulation**.** Shown are observed (H_O_) and expected (H_S_) heterozygosities, inbreeding coefficient (G_IS_) according to Nei’s statistics (1987), and p-value.

|  | **Subpopulation** | **H_O_** | **H_S_** | **G_IS_** | **p-value** |
| --- | --- | --- | --- | --- | --- |
| Poor |  | 0.489 | 0.574 | 0.147 | <0.001 |
|  | *Females* | 0.461 | 0.523 | 0.118 | <0.05 |
|  | *Fighters* | 0.356 | 0.542 | 0.342 | <0.001 |
|  | *Scramblers* | 0.583 | 0.603 | 0.032 | 0.306 |
| Rich |  | 0.402 | 0.615 | 0.346 | <0.001 |
|  | *Females* | 0.361 | 0.492 | 0.267 | <0.001 |
|  | *Fighter* | 0.329 | 0.592 | 0.444 | <0.001 |
|  | *Scramblers* | 0.497 | 0.605 | 0.178 | <0.01 |

Table S2.3 Pairwise F_ST_ values for population differentiation. Shown are the genetic differentiation values per subpopulation, Poor (P), and Rich (R) environments, Female (F), Male Fighter (MF), and Male Scrambler (MS) subpopulations. Significant differences are represented by * after Bonferroni correction.

|  | **P_F** | **P_MF** | **P_MS** | **R_F** | **R_MF** | **R_MS** |
| --- | --- | --- | --- | --- | --- | --- |
| **P_F** | -- |  |  |  |  |  |
| **P_MF** | 0.056* | -- |  |  |  |  |
| **P_MS** | 0.025 | 0.044* | -- |  |  |  |
| **R_F** | 0.286* | 0.165* | 0.223* | -- |  |  |
| **R_MF** | 0.076* | 0.095* | 0.104* | 0.223* | -- |  |
| **R_MS** | 0.167* | 0.082* | 0.122* | 0.080* | 0.090* | -- |

Table S2.4 Summary of hierarchical AMOVA. Shown are standard deviation (jack-knifing over loci), % of variation, and values of the F-statistic on different levels (between environment, among subpopulation within environment, among individuals within subpopulation, and within individuals), with their corresponding F and p-values. F_CT_ = the proportion of total variance that results from genetic differences among groups, F_SC_ = the proportion of variance among subpopulations within clusters, F_IS_ = the proportion of variance among individuals within subpopulation, F_IT_ = the proportion of variance among individuals within the total population

| **Variance component** | **SD** | **Variation (%)** | **Statistic** | **F-value** | **p-value** |
| --- | --- | --- | --- | --- | --- |
| *Between environment* | 0.036 | 0.072 | F_CT_ | 0.072 | 0.191 |
| *Among subpopulations in environment* | 0.083 | 0.078 | F_SC_ | 0.089 | <0.001 |
| *Among individuals in subpopulation* | 0.112 | 0.175 | F_IS_ | 0.207 | <0.001 |
| *Within individuals* | 0.120 | 0.670 | F_IT_ | 0.330 | <0.001 |

Table S2.5 Genetic differentiation between strategies (fighters and scramblers) compared to total population within that environment (rich and poor). The F_ST_ values of the different ARTs within environment compared to the rest of the subpopulation:

| **Environment** | **F_ST_ value** | **p-value** |
| --- | --- | --- |
| Poor *Fighter*  *Scrambler* | 0.044  0.019 | <0.001  <0.05 |
| Rich  *Fighter*  *Scrambler* | 0.142  0.025 | <0.001  <0.05 |
