# Supplementary material for: The role of genetic diversity in the evolution and maintenance of environmentally-cued, male alternative reproductive tactics
Source: BMC Evol Biol. 2019 Feb 18;19:58. doi: 10.1186/s12862-019-1385-4 (PMC6379956; doi:10.1186/s12862-019-1385-4)
Supplement: Supplementary file 2 — Table S1.1. Allele frequencies by sex, morph, and environments. (DOCX 39 kb) [file 12862_2019_1385_MOESM2_ESM.docx]

Additional File 2

Table S1.1 Allele frequencies by sex, morph, and environments. When an allele is absent from a category, it is represented by - , private alleles by sex are bolded, and alleles restricted by environment are denoted by *.

|  |  |  |  | **Rrms18** |  |  |  |  |
| --- | --- | --- | --- | --- | --- | --- | --- | --- |
|  | *Rich* |  |  |  | *Poor* |  |  |  |
|  | Female | Male | Scrambler | Fighter | Female | Male | Scrambler | Fighter |
| Alleles | N=102 | N=112 | N=66 | N=46 | N=58 | N=146 | N=60 | N=86 |
| 130 | 0.059 | 0.089 | 0.152 | - | 0.017 | 0.075 | 0.067 | 0.081 |
| **141*** | - | 0.134 | 0.106 | 0.174 | - | - | - | - |
| 142 | 0.902 | 0.554 | 0.621 | 0.457 | 0.483 | 0.658 | 0.600 | 0.698 |
| 143 | 0.039 | 0.223 | 0.121 | 0.370 | 0.500 | 0.267 | 0.333 | 0.221 |
|  |  |  |  | **Rrms34** |  |  |  |  |
|  | *Rich* |  |  |  | *Poor* |  |  |  |
|  | Female | Male | Scrambler | Fighter | Female | Male | Scrambler | Fighter |
| Alleles | N=98 | N=128 | N=84 | N=44 | N=54 | N=134 | N=60 | N=74 |
| **106*** | - | - | - | - | - | 0.015 | - | 0.027 |
| 121 | 0.061 | 0.039 | 0.060 | - | 0.056 | 0.060 | 0.050 | 0.068 |
| 122* | - | - | - | - | 0.037 | 0.022 | 0.017 | 0.027 |
| 123 | 0.071 | 0.055 | 0.083 | - | 0.185 | 0.149 | 0.133 | 0.162 |
| 124* | - | - | - | - | 0.019 | 0.037 | 0.083 | - |
| **131** | - | 0.156 | 0.131 | 0.205 | - | 0.045 | - | 0.081 |
| 132 | 0.480 | 0.336 | 0.333 | 0.341 | 0.241 | 0.269 | 0.283 | 0.257 |
| 133 | - | 0.141 | 0.083 | 0.250 | 0.019 | 0.037 | 0.017 | 0.054 |
| 134 | 0.378 | 0.188 | 0.226 | 0.114 | 0.037 | 0.067 | 0.033 | 0.095 |
| 135 | 0.010 | 0.086 | 0.083 | 0.091 | 0.407 | 0.291 | 0.367 | 0.230 |
| **136*** | - | - | - | - | - | 0.007 | 0.017 | - |
|  |  |  |  | **Rrms40** |  |  |  |  |
|  | *Rich* |  |  |  | *Poor* |  |  |  |
|  | Female | Male | Scrambler | Fighter | Female | Male | Scrambler | Fighter |
| Alleles | N=100 | N=140 | N=78 | N=62 | N=58 | N=126 | N=60 | N=66 |
| 85* | 0.030 | 0.014 | - | 0.032 | - | - | - | - |
| **86*** | - | - | - | - | - | 0.016 | - | 0.030 |
| **98*** | - | - | - | - | - | 0.008 | 0.017 | - |
| 105 | 0.130 | 0.071 | 0.103 | 0.032 | 0.069 | 0.008 | 0.017 | - |
| 106 | 0.280 | 0.221 | 0.218 | 0.226 | 0.707 | 0.746 | 0.767 | 0.727 |
| **108*** | - | 0.007 | 0.013 | - | - | - | - | - |
| **109** | - | 0.007 | - | 0.016 | - | 0.008 | 0.017 | - |
| 117 | 0.520 | 0.250 | 0.295 | 0.194 | 0.034 | - | - | - |
| 118 | 0.040 | 0.429 | 0.372 | 0.500 | 0.190 | 0.214 | 0.183 | 0.242 |
|  |  |  |  | **Rrms44** |  |  |  |  |
|  | *Rich* |  |  |  | *Poor* |  |  |  |
|  | Female | Male | Scrambler | Fighter | Female | Male | Scrambler | Fighter |
| Alleles | N=90 | N=142 | N=82 | N=60 | N=60 | N=100 | N=60 | N=40 |
| 91 | 0.067 | 0.183 | 0.232 | 0.117 | 0.250 | 0.330 | 0.417 | 0.200 |
| **97*** | 0.244 | - | - | - | - | - | - | - |
| 98 | 0.689 | 0.817 | 0.768 | 0.883 | 0.750 | 0.670 | 0.583 | 0.800 |

|  |  |  |  | **Rrms72** |  |  |  |  |
| --- | --- | --- | --- | --- | --- | --- | --- | --- |
|  | *Rich* |  |  |  | *Poor* |  |  |  |
|  | Female | Male | Scrambler | Fighter | Female | Male | Scrambler | Fighter |
| Alleles | N=88 | N=140 | N=80 | N=60 | N=62 | N=94 | N=60 | N=34 |
| 128 | 0.205 | 0.157 | 0.175 | 0.133 | 0.274 | 0.287 | 0.183 | 0.417 |
| 129 | 0.011 | 0.036 | - | 0.083 | - | 0.032 | 0.050 | - |
| 136 | 0.443 | 0.436 | 0.475 | 0.383 | 0.419 | 0.319 | 0.350 | 0.265 |
| 137 | 0.023 | 0.057 | - | 0.133 | - | 0.032 | 0.050 | - |
| **138*** | - | - | - | - | - | 0.011 | - | 0.029 |
| 140 | 0.284 | 0.293 | 0.350 | 0.217 | 0.306 | 0.245 | 0.267 | 0.206 |
| **141*** | - | - | - | - | - | 0.064 | 0.100 | - |
| 142 | 0.034 | 0.021 | - | 0.050 | - | 0.011 | - | 0.029 |

|  |  |  |  | **Rrms91** |  |  |  |  |
| --- | --- | --- | --- | --- | --- | --- | --- | --- |
|  | *Rich* |  |  |  | *Poor* |  |  |  |
|  | Female | Male | Scrambler | Fighter | Female | Male | Scrambler | Fighter |
| Alleles | N=92 | N=140 | N=82 | N=58 | N=60 | N=106 | N=60 | N=46 |
| 84 | 0.141 | 0.157 | 0.220 | 0.069 | 0.117 | 0.226 | 0.267 | 0.147 |
| 85 | - | 0.014 | 0.012 | 0.017 | 0.033 | 0.075 | 0.133 | - |
| 91 | 0.761 | 0.443 | 0.646 | 0.155 | 0.033 | 0.236 | 0.100 | 0.413 |
| 92 | 0.098 | 0.386 | 0.122 | 0.759 | 0.817 | 0.462 | 0.500 | 0.413 |
